# Supplementary material for: Pivotal Role of the Granularity Uniformity of the WO3 Film Electrode upon the Cyclic Stability during Cation Insertion/Extraction
Source: Nanomaterials (Basel). 2023 Mar 8;13(6):973. doi: 10.3390/nano13060973 (PMC10057934; doi:10.3390/nano13060973)
Supplement: Supplementary file 1 [file nanomaterials-13-00973-s001.zip › nanomaterials-2243219-supplementary.pdf]

nanomaterials

Supplementary Materials

Pivotal Role of the Granularity Uniformity of the WO_3 Film Electrode upon the Cyclic Stability during Cation Insertion/Extraction

Zhaocheng Zhang [1], Haoyuan Chen [1], Zicong Lin [1], Xiongcong Guan [1], Jiong Zhang [2] ,Xiufeng Tang [1,3,*], Yunfeng Zhan [1,3] and Jianyi Luo [1,3,*]

1 School of Applied Physics and Materials, Wuyi University, Jiangmen 529020, China; supremezzc@sina.cn (Z.Z.); 13822465993@163.com (H.C.); 15812365010@163.com (Z.L.); guanxc002719@163.com (X.G.), zhanyf6@163.com (Y.Z.)
2 School of Civil Engineering and Architecture, Wuyi University, Jiangmen 529020, China; jiongzhang@wyu.edu.cn
3 Research Center of Flexible Sensing Materials and Device Application Technology, Wuyi University, Jiangmen 529020, China
* Correspondence: tbrenda@sina.com (X.T.); luojiany@mail3.sysu.edu.cn (J.L.)

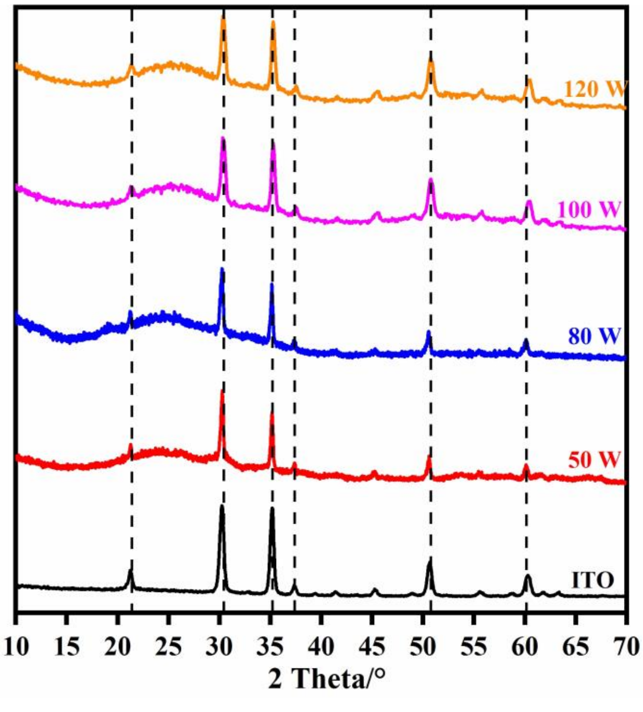

Figure S1. XRD spectra of the prepared WO_3 films.

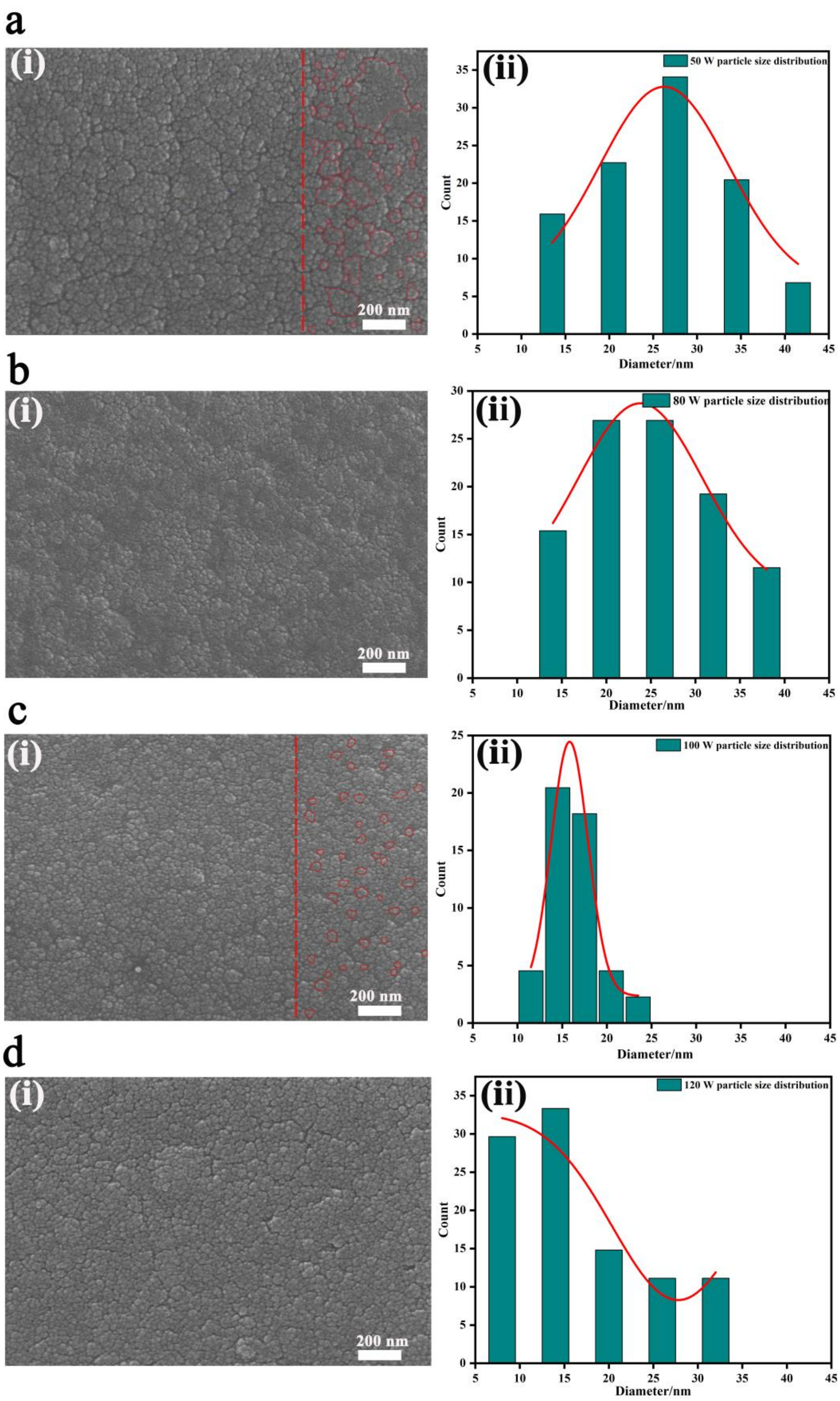

Figure S2. SEM images of the prepared WO_3 films (i) and corresponding particle size distribution analysis through Nano Measure software (ii), **a** 50 W; **b** 80 W; **c** 100 W; **d** 120 W, where red-line circles in **a** and **c** pictured the particle size distribution.

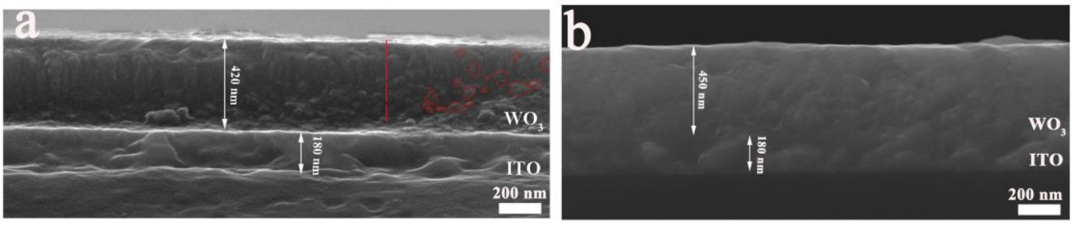

Figure S3. Cross-sectional SEM images of the as-prepared WO_3 films, **a** 50 W; **b** 100 W, where red-line circles in **a** pictured the particle size distribution.

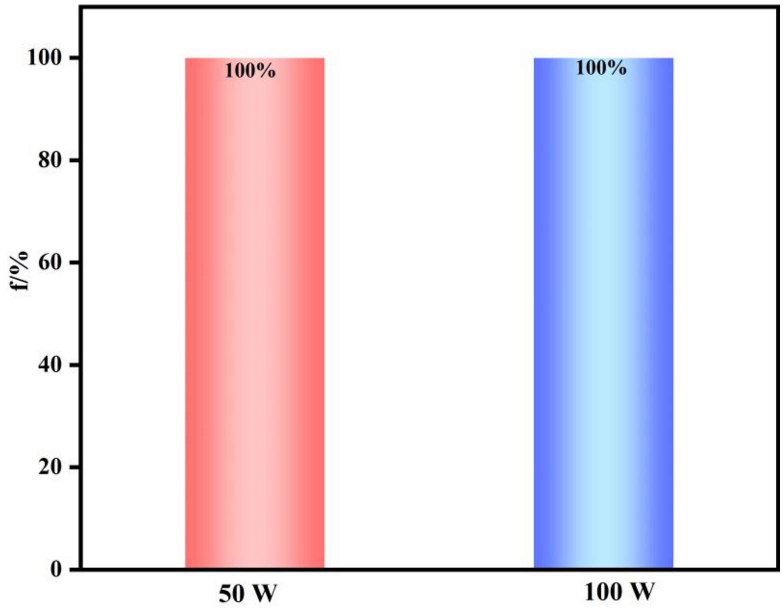

Figure S4. Adhesive force analysis of the as-prepared WO_3 films with ITO substrates.

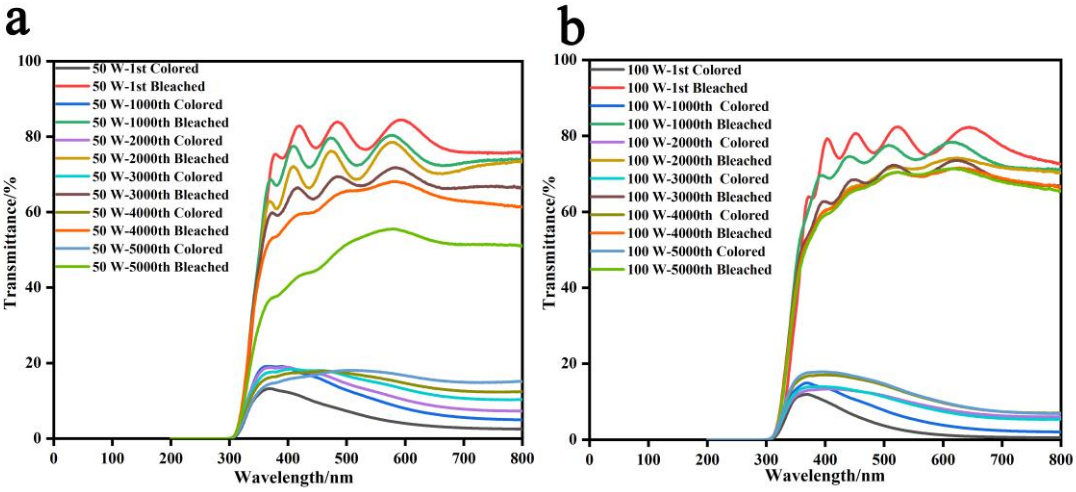

Figure S5. Evolution of transmittance spectra of the WO_3 films during 5000 cyclic voltammetry (CV) cycles, **a** 50 W; **b** 100 W.

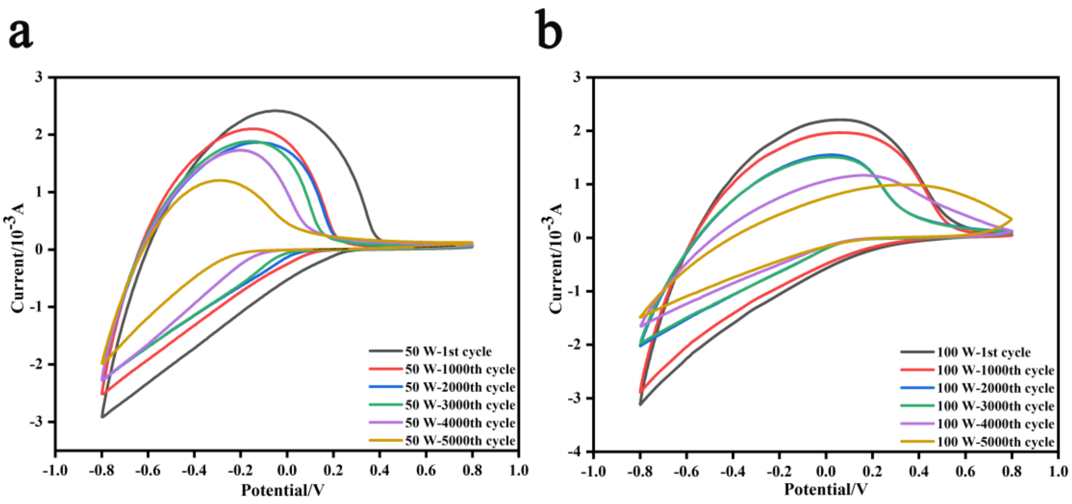

Figure S6. Evolution of CV curves of the WO_3 films during 5000 CV cycles at the scanning rate of 0.1 V/s versus Ag/AgCl, **a** 50 W; **b** 100 W.

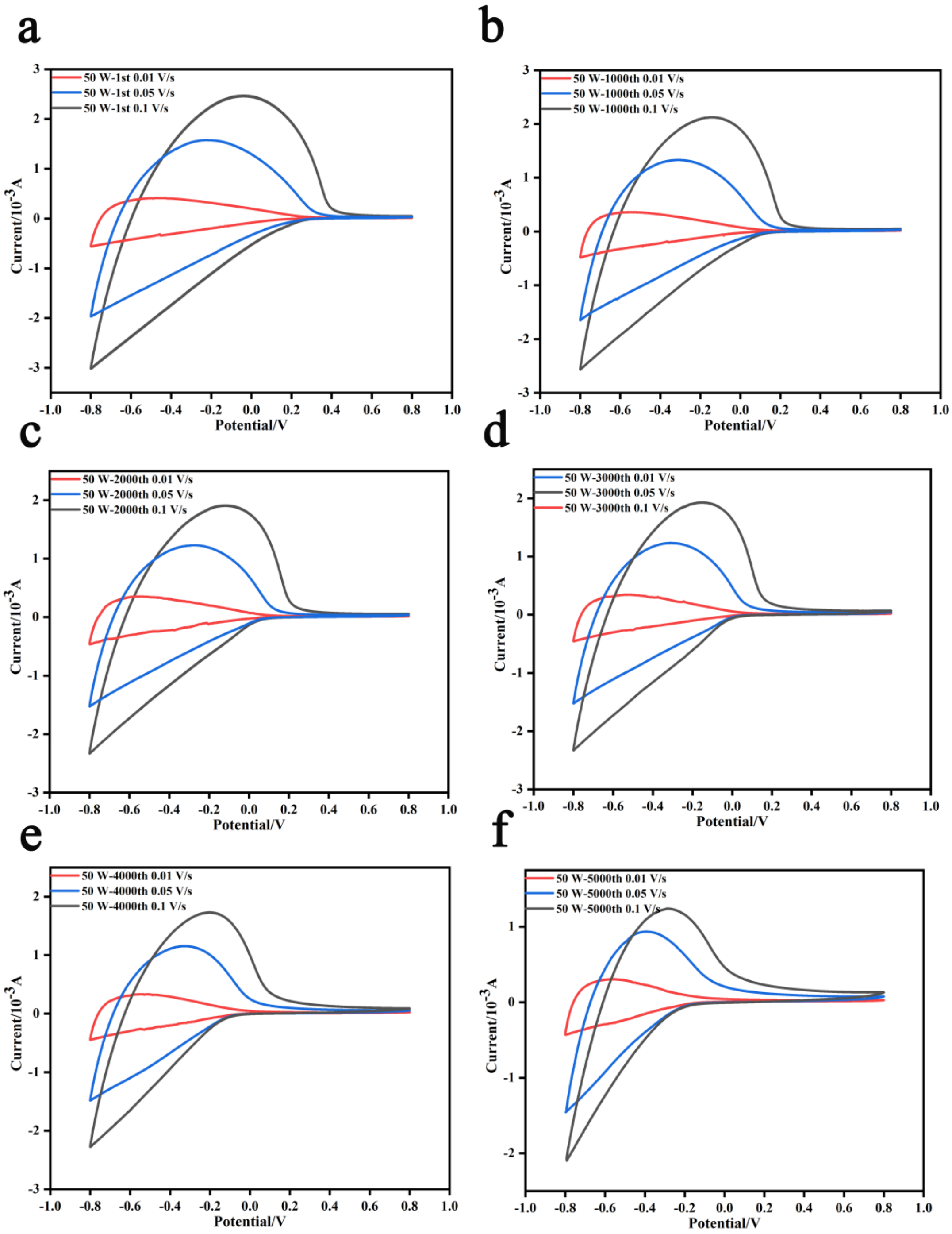

Figure S7. CV curves of the 50W-deposited WO_3 film during 5000 CV cycles at scanning rates ranging from 0.01 to 0.1 V/s versus Ag/AgCl, **a** the first cycle; **b** the 1000th cycle; **c** the 2000th cycle; **d** the 3000th cycle; **e** the 4000th cycle; **f** the 5000th cycle.

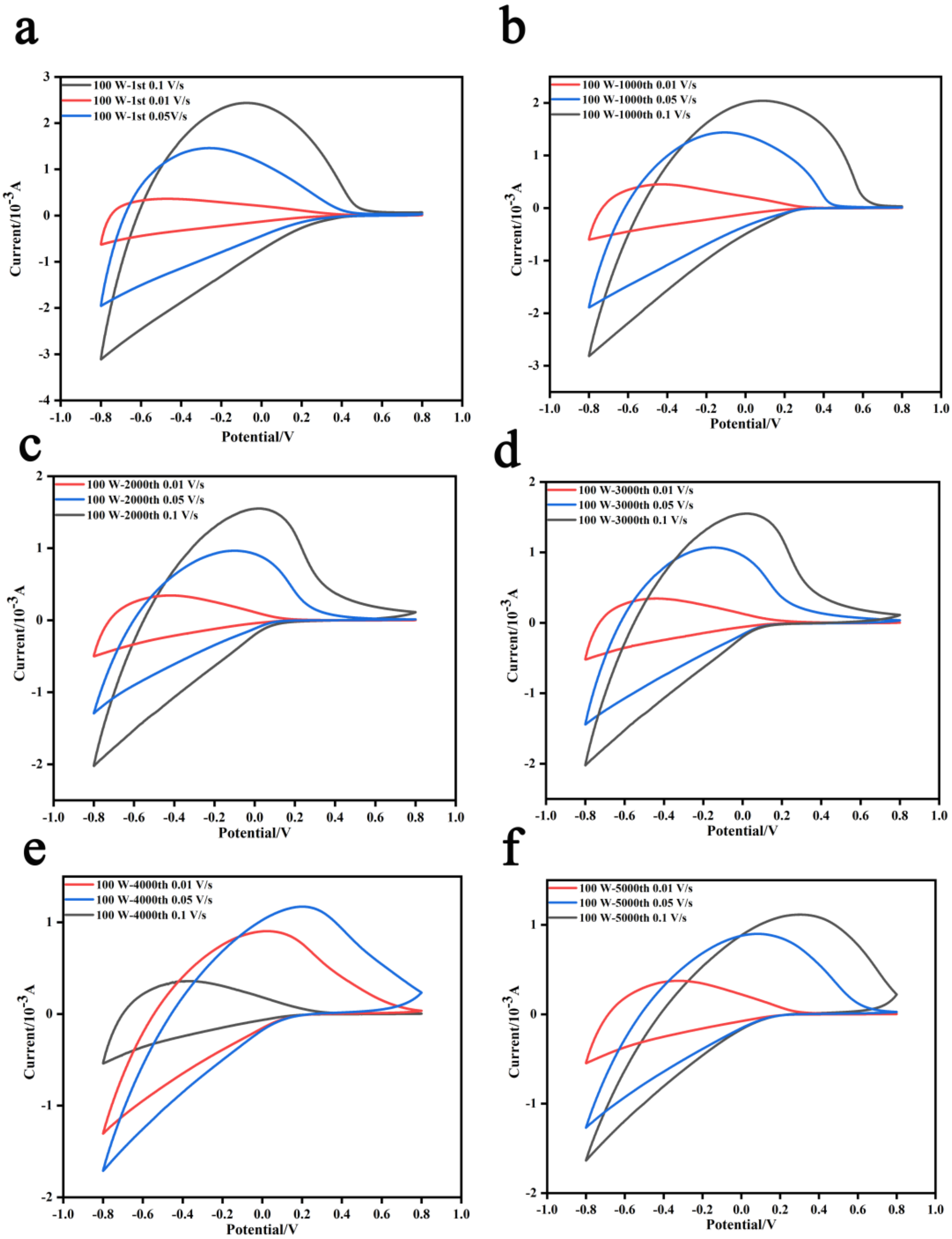

Figure S8. CV curves of the 100W-deposited WO_3 film during 5000 CV cycles at scanning rates ranging from 0.01 to 0.1 V/s versus Ag/AgCl, **a** the first cycle; **b** the 1000th cycle; **c** the 2000th cycle; **d** the 3000th cycle; **e** the 4000th cycle; **f** the 5000th cycle.

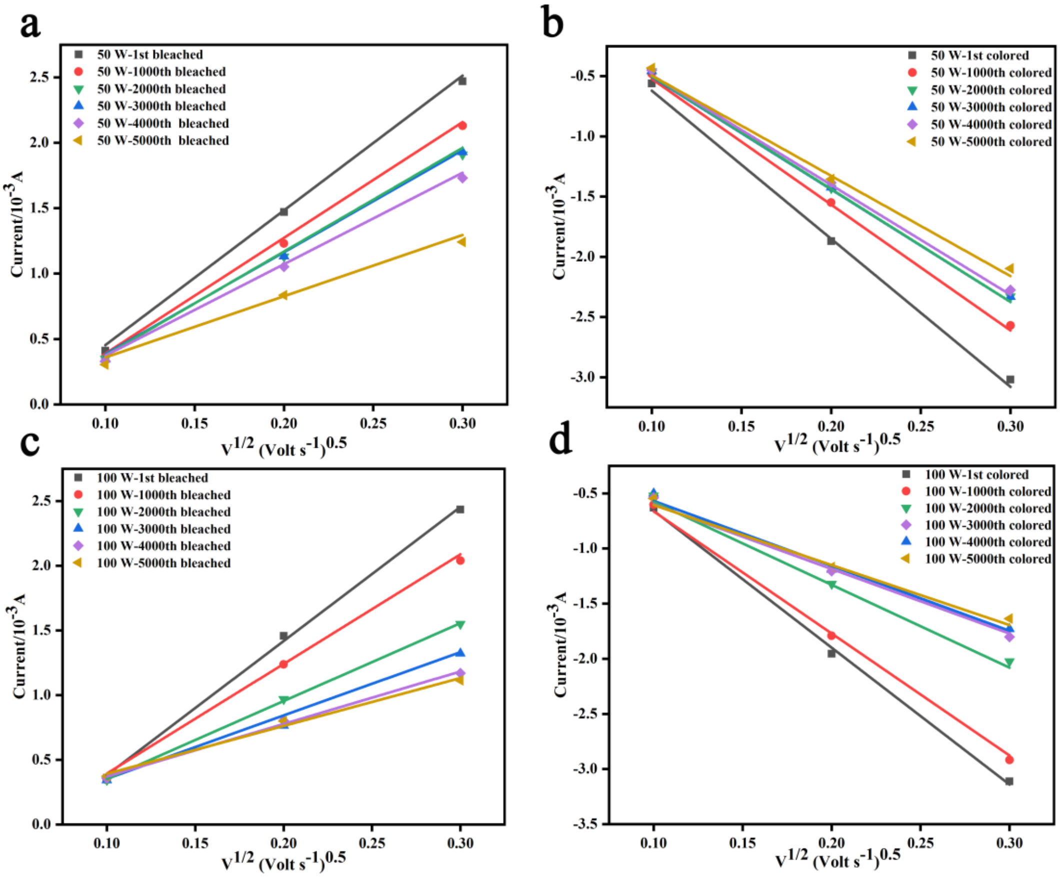

Figure S9. Evolution of the redox peaks versus square root of the scanning rate, **a** in bleaching process of the 50W-deposited film; **b** in coloring process of the 50W-deposited film; **c** in bleaching process of the 100W-deposited film; **d** in coloring process of the 100 W-deposited film.
